# Echoes of the Month

**Published:** 1924-12

**Authors:** 


### ECHOES OF THE MONTH.

### THE COST OF RHEUMATISM.

Dr. James R. Kerr, surgeon-in-charge of the Pilkington Special Hospital, St. Helens, in a lecture on "Chronic Joint Diseases," at the Royal Institution of Public Health, Russell Square, stated that there is an annual cost of nearly one million pounds in sick benefit paid by the approved societies and a national loss per year of over a million and a-half weeks' work due to invalidity from chronic joint cases resulting from rheumatic diseases. Attacks of "rheumatic fever," he said, diminished after the age of forty-five, while most chronic joint cases were over that age, and of those 80 per cent. had no history of previous attacks of "acute rheumatism." He gave remarkable instances of restoration of victims to relative action and comfortable wage-earning life by physiotheraphy on orthopædic lines and post operative orthopædic treatment, and by a series of lantern slides illustrated ingenious appliances and methods employed to restore power of movement to affected joints.

### A MUNICIPAL SOLARIUM.

The Bermondsey Borough Council have decided to erect a municipal solarium as one of the measures in their campaign for the prevention of tuberculosis. Negotiations are already taking place to obtain a piece of land about three-quarters of an acre in extent, where it is proposed to erect a solarium. This would consist of a wooden shed with a verandah and a concrete slope in front which could be used for exposing patients to the fresh air and sun when this is available. It is also proposed to erect sixteen arc lamps, which could be used for artificial sunlight treatment when natural sunlight is not available. The remainder of the ground would be turfed and used for giving children physical exercises in the sun. Lessons in elementary hygiene would be imparted at the same time.

### A RECORD NUMBER OF OPERATIONS.

A world record that is as unusual as it is unpleasant, to use the mildest term, is claimed (says the *Manchester Guardian*) by Harry B. Smythe, of St. Louis, who has just had his 134th operation under an anæsthetic. The sufferer is fifty years of age, and he was first under the surgeon's knife when he was ten. A case of typhoid fever was followed by a bone infection, and it was necessary for a surgeon to scrape away the infected portion. A new bone grew in its place, but Smythe was found to be a victim of osteomyelitis, a virulent bone disease. In the next thirteen years he had to undergo 115 operations, of which he has kept an accurate record. It seemed as if the fight waged against the disease during those thirteen years was successful, as Smythe was able to occupy a place in the world until 1916, when he again had to go into hospital, where he remained for a year because of the old complaint, the total of his operations by the end of that time amounting to 133. Recently he had again to go to hospital, and was told that the 134th operation would be necessary for bone infection, and would have to be followed by another one to relieve an injury he suffered in an accident last year.

### REPLACING NATURAL ENVIRONMENT.

"Cities," said Dr. Veitch Clarke in an address at the Collyhurst Recreation Hall recently, "had entirely abolished natural environment." The Mersey School endeavoured to replace the countryside and quiet home. Even when it was set in the midst of a deadly dull and hard-working centre like Collyhurst, where were no green fields, no beautiful rivers, no deep quiet of the countryside, it had at least a long verandah where the children could lie out asleep, and there were natural pictures, natural toys, and the means of conducting the minds and characters of the children. The attendance of a child at a welfare centre and a nursery school meant a weekly visit of the mother to the centre, and the school meant the daily training of the child, and, with all respect, of the mother also.

### A GYMNASIUM FOR INFANTS.

A gymnasium for infants in arms has been opened in Berlin by Major A. D. Detleff Neumann-Neurode, formerly physical instructor in a military school at Potsdam. "Every six-months-old baby should do five to ten minutes' daily exercises with its mother or nurse," said Major Neumann-Neurode in an interview. "If my advice were followed, the appalling number of cripples in this country would be greatly diminished. Regular, graduated exercises ensure harmonious development, correct posture and firm bones." The gymnasium, with its furnishings of miniature swings, ladders and bars, is for the professor's older pupils—that is, those from eighteen months to five years old.

### SURGERY AND CANCER.

Some examples of the progress of modern surgery were given at the Clinical Congress of the American College of Surgeons held recently in New York. Dr. H. B. Devine, of Melbourne, Australia, held the centre of interest with his revelation of a simple method of removing ulcers from the stomach, which medical experts say is likely to revolutionise stomach surgery. Instead of dangerous cutting by high-priced specialists, Dr. Devine said an ordinary surgeon may remove cancers from the stomach by merely severing the diseased part and sympathetic nerves. Modern civilisation was responsible for most gastric complaints, Dr. Devine said, because of its disturbing effects upon the nervous system. The sympathetic nervous system was a thing upon which we took our daily strain of living, he declared, and when it was worn and ragged it became diseased. By simply removing the sympathetic nerves controlling the stomach, Dr. Devine claimed that the organ was permitted to function independently and was not affected by mental agitations.

### CARBON MONOXIDE POISONING IN SMALL GARAGES.

The Surgeon-General of the United States Public Health Service warns automobile owners of the danger involved in running a gasoline engine in a small closed space for any considerable period of time. A man was recently found dead in a garage at Baltimore, the engine of his car still running and giving off a small proportion of carbon monoxide The attack of carbon monoxide poisoning comes on insidiously, and consciousness is gradually lost. Even though the victim may become aware of the danger, he is often unable to escape from it because of the great loss of motor power. The automobile worker in a small garage is most frequently the victim. It therefore behoves every person who runs his engine in a small garage to see to it that the room is properly ventilated by having the windows and doors opened if he expects to run the engine for even a few minutes.

### SEAWATER CURE.

In a lecture on "The Curative Properties of Sea Water," at the London College of Physiology, Dr. Thomas E. Lawson stated that the people of Jamaica, on returning to their villages from the sea, take with them a quantity of the water. "A patient from Ceylon told me this summer," he said, "that on leaving Colombo his daughter was desperately sea-sick for some days. A coloured sailor, noticing her condition, said: 'Let missie drink sea-water.' As the doctor could not stop the sickness, he resolved to try his remedy, and after she had sipped it for a time the sickness was stopped. A small dose of sea-water each day kept the child from a return of the trouble throughout the voyage." Sea-water treatment, said the lecturer, is carried out by means of hypodermic injections. "The most fascinating province in which sea-water works miracles," concluded Dr. Lawson, "is in treating the unborn child that it may not suffer from the sins of its father, and a healthy child may be born."

### INFECTION BY EATING GRASS.

The habit of nibbling blades of grass while on walking tours may, it is stated, have caused the death of a man in one of the London hospitals. He suffered from actinomycosis, and it was discovered that he was a great walker, and often used to eat grass as he went along. During the last year he underwent five operations but without avail. Actinomycosis is an infection by a fungus. It especially affects horned cattle and pigs, and, in man, causes abscesses, inflammation, and the formation of tumours. A great deal is known about it, and cases are fairly common in hospital.

